# Deep learning-based speech analysis for Alzheimer’s disease detection: a literature review

**DOI:** 10.1186/s13195-022-01131-3

**Published:** 2022-12-14

**Authors:** Qin Yang, Xin Li, Xinyun Ding, Feiyang Xu, Zhenhua Ling

**Affiliations:** 1 NELSLIP, University of Science and Technology of China, Hefei, China; 2 iFlytek Research, iFlytek Co.Ltd, Hefei, China

**Keywords:** Alzheimer’s disease detection, Speech analysis, Deep learning

## Abstract

**Background:**

Alzheimer’s disease has become one of the most common neurodegenerative diseases worldwide, which seriously affects the health of the elderly. Early detection and intervention are the most effective prevention methods currently. Compared with traditional detection methods such as traditional scale tests, electroencephalograms, and magnetic resonance imaging, speech analysis is more convenient for automatic large-scale Alzheimer’s disease detection and has attracted extensive attention from researchers. In particular, deep learning-based speech analysis and language processing techniques for Alzheimer’s disease detection have been studied and achieved impressive results.

**Methods:**

To integrate the latest research progresses, hundreds of relevant papers from ACM, DBLP, IEEE, PubMed, Scopus, Web of Science electronic databases, and other sources were retrieved. We used these keywords for paper search: (Alzheimer OR dementia OR cognitive impairment) AND (speech OR voice OR audio) AND (deep learning OR neural network).

**Conclusions:**

Fifty-two papers were finally retained after screening. We reviewed and presented the speech databases, deep learning methods, and model performances of these studies. In the end, we pointed out the mainstreams and limitations in the current studies and provided a direction for future research.

## Background

Dementia is the most common neurodegenerative disease among the elderly, of which Alzheimer’s disease (AD) is the most common type. According to data from the World Health Organization, the current incidence of AD has shown a significant upward trend in recent years, and the number of patients will reach 152 million in 2050 [1], which will affect the health of the people seriously and cause an enormous economic burden on home care and social security. However, effective treatment is not yet available. Studies have shown that the early diagnosis and intervention based on early assessment and screening of cognitive impairment can help maintain healthy brain activity, retard irreversible brain decline, delay disease progression, and prolong patient life [2]. In this case, early detection of mild cognitive impairment (MCI), which is the early stage of AD, is very important for delaying cognitive state decline.

Currently, the mainstream clinical methods for AD detection include scale testing, brain magnetic resonance imaging measurement (MRI), cerebrospinal fluid analysis, etc. These methods are either time-consuming and labor-intensive, or expensive and unfriendly to subjects’ experience. In general, traditional AD detection methods such as magnetic resonance imaging, positron emission tomography (PET) imaging, and cerebrospinal fluid (CSF) assays [3], are not appropriate for large-scale nationwide early AD screening applications. Therefore, some studies focus on developing a cheaper and more convenient method to detect AD.

Relevant studies have shown that language disorders usually appear in the early process of AD, and it is possible to detect AD by capturing the acoustic and linguistic features of subjects through audio and automatic speech recognition technology [4–6]. Some studies have given the results of studies on distinguishing characteristics between AD and healthy control (HC) group. Compared with cognitive normal people, AD patients usually speak more slowly with more pauses between words [7] and suffer from word finding and word retrieval difficulties [6, 8, 9].

Dozens of speech-based methods have been explored for the research on AD detection. Studies have shown that the acoustic measures have a high correlation with pathological language features and voice changes in automatic language processing were proven to be useful for AD detection [10, 11]. In addition, previous studies of speech pathology have revealed that people with dementia have linguistic manifestations including pauses, filler words, restarts, repetitions, and incomplete statements. Fraser, K.C. et al. extracted linguistic features such as semantics, syntax, and information and achieved 91% accuracy [4] in the AD detection task by using the logistic regression classifier. Liu, Z. et al. extracted and fused duration features, acoustic features, linguistic features, the AD detection, and linguistic features, and finally obtained 81.9% accuracy of AD detection based on the logistic regression classification method [12]. In addition to these, Satt, A. et al. utilized recordings while subjects completed cognitive tasks to extract relevant acoustic features, and achieved an accuracy of 87% in the classification between AD and control [5].

With the wide application of deep learning, we can find that neural networks have made significant progress in the field of speech modeling. Hinton, G. et al. applied deep neural networks (DNNs) to acoustic modeling and obtained better recognition results than Gaussian Mixed Model (GMM), thus opening up a new field in speech recognition [13]. Therefore, researchers began to try to apply various deep learning methods to the field of speech-based AD detection. Rosas, D.S. et al. extracted linguistic features and used a 3-layer neural network reaching a binary classification accuracy of 78.3% [14]. However, there is fewer speech data for Alzheimer’s patients, and the improvement in classification results is relatively small by using neural networks. Recent studies have shown that pre-trained models such as BERT [15] achieve promising results on a variety of benchmark tasks, and can capture a wide range of linguistic facts including lexical knowledge, phonology, syntax, semantics, and pragmatics without a lot of data. Apart from this, the pre-trained automatic speech recognition (ASR) model can not only get the transcribed text of speech but also extract acoustic embeddings which can be used to represent the conversion in speech for better automatic analysis. Toth, L. et al. obtained phonetic segmentation and label of the input signal by applying an ASR model based on a special convolutional deep neural network, thereby obtaining acoustic features such as speech rate, pause, and hesitation rate [16]. Judging by the current research trends, the deep learning method is the most mainstream method for AD detection now.

Simultaneously, some review papers on AD detection have also been published, such as a systematic review about speech-based detection and classification of AD written by Inês Vigo et al. [17]. However, most of the classification methods are based on traditional machine learning methods, which have certain limitations due to the excellent performance achieved by deep learning methods in AD detection.

Therefore, this paper focuses on deep learning-based speech analysis for AD detection. This research paper is organized as follows: the objects of this review in the “Objectives” section, the search and selection process is introduced in the “Materials and methods” section, the results in the “Results” section, the discussion of these selected papers in the “Conclusions” section, and the limitation of our work and our future goals in the “Discussions” section.

## Objectives

To make a comprehensive discussion on the current application of deep learning in speech-based AD detection, this review conducted a systematic analysis of selected papers in response to the following 5 questions:

1. What were the characteristics of the databases involved in reported studies?
2. What deep learning model architectures were included in reported studies?
3. How were these deep learning model architectures used in reported studies?
4. What classification performance has been achieved?
5. What were the mainstreams and limitations of reported studies?

## Materials and methods

### Search process

**Fig. 1:**
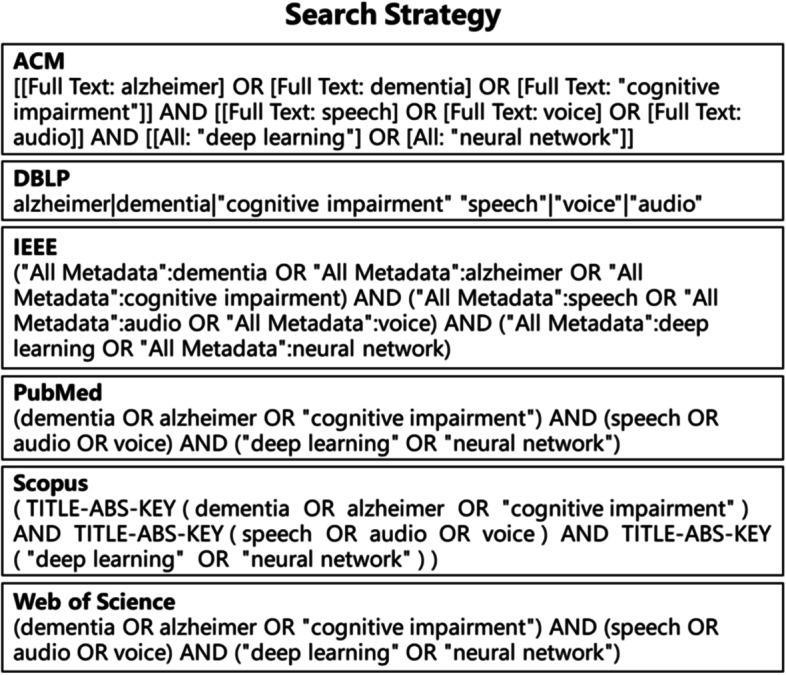
Search strategies in ACM, DBLP, IEEE, PubMed, Scopus, and Web of Science

Our searches were conducted on the following electronic databases: ACM, DBLP, IEEE, PubMed, Scopus, and Web of Science. Unlike most previous review papers on “Alzheimer’s disease detection” [18], we paid more attention to these papers which used deep learning methods to analyze speech data of elderly people in different health states (AD, MCI, and HC). Therefore, we used the following keywords for paper search: (Alzheimer OR dementia OR cognitive impairment) AND (speech OR voice OR audio) AND (deep learning OR neural network). Figure 1 listed all the search strategies. The last search was conducted on 19 January 2022.

### Selection process

The exclusion criteria were as follows: (1) studies that did not use deep learning methods; (2) studies do not focus on speech or text data; (3) studies without a group of MCI and AD; (4) papers that were not written in English; (5) studies cannot find the full text. Initial study selection was performed by two reviewers independently. To minimize the bias in selecting studies, papers that were not sure to include were resolved in a discussion with the third reviewer.

### Data extraction and synthesis

The analyzed data in our studies include database names, task types, language types, label distributions, and whether the databases include an audio or corresponding transcript or not.

## Results

### Study selection

**Fig. 2:**
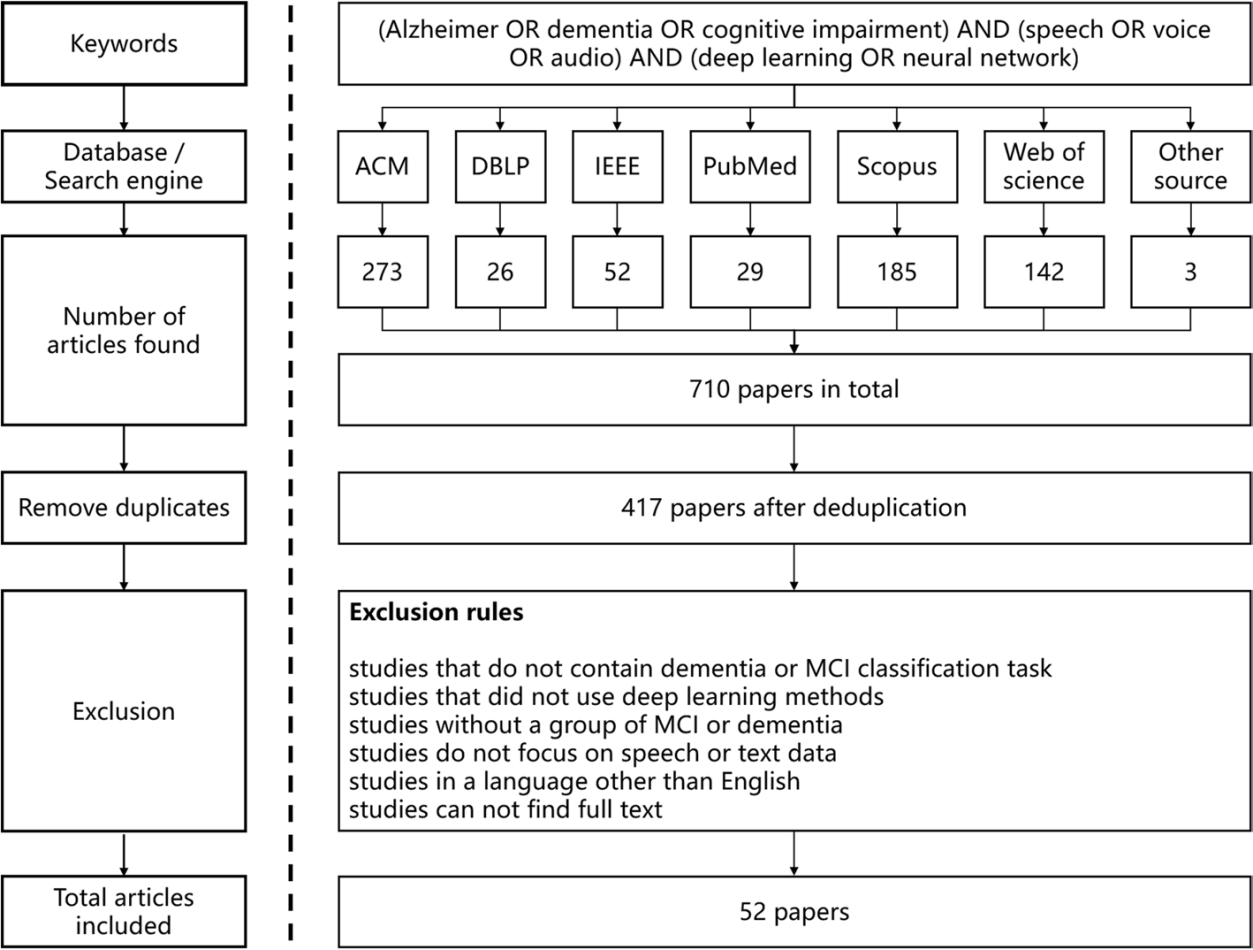
Flow diagram of the search and selection process in our study

The detail of our search process is displayed in Fig. 2 through a flow diagram. other source papers retrieved from the ADReSS website [19] which were not found in the other six sources. After the search process, a total of 710 papers were retrieved; 293 duplicates were removed by Endnote and manual screening. After screening by our exclusion rules, 52 studies were finally included.

### Speech databases

In the process of building a deep learning model, a high-quality database can improve the quality of model training and the accuracy of prediction. At present, several speech databases for cognitive impairment of the elderly have been established around the world, providing great support for researchers to explore more efficient cognitive impairment assessments.

According to our research, the linguistic tasks involved in the studies on AD detection based on deep learning methods can be divided into three categories: semantic verbal fluency (SVF), spontaneous speech (SS), and reading. Therefore, the related speech databases will also be introduced from these three aspects in this review (Table 1).

**Table 1:** Dementia-related speech-based databases information

Task name			Database name	Abbreviation	Language	Label distribution	Speech included	Transcription included
VF	SVF	Animal naming	PGA-OREKA [20]	VF1	Spain	HC (62)/MCI (38)	Yes	No
			- [21]	VF2	French, Dutch, and German	HC (66)/MCI (66)	Yes	-
		Vegetable naming	Mandarin_Lu (DementiaBank) + NTU dataset [22]	VF3	Chinese	AD (30)/HC (30)	Yes	No
		Location naming	Mandarin_Lu (DementiaBank) + NTU dataset [22]	VF4	Chinese	AD (30)/HC (30)	Yes	No
SS	Conversation/interview		PROMPT database [23]	cvs1	Japanese	Dementia (49)/MCI(42)/HC(72)	Yes	-
			- [24]	cvs2	Italian	eD (16)/MCI (32)/HC (48)	Yes	Yes
			The Carolina Corpus Conversation database [14]	cvs3	English	AD (30)/HC (16)	Yes	Yes
			IVA dataset [25]	cvs4	English	ND (21)/MCI (24)/HC (25)	Yes	-
			The Hungarian MCI-mAD Database [26, 27]	cvs5	Hungarian	MCI (48)/HC (36) AD (25)/MCI (25)/HC (25)	Yes	No
			AZTIAHO AZTIAHORE [28]	cvs6	multilingual	AD (20)/HC (50) AD (20)/MCI (20)	Yes	No
			- [29]	cvs7	Italian	MCI (19)/HC (20)	Yes	Yes
			- [16]	cvs8	Hungarian	MCI (32)/HC (19)	Yes	No
			Framingham Heart Study Dataset [30]	cvs9	English	Dementia (223)/MCI (309)/HC (291)	Yes	No
	Recall		NTUHV dataset [31]	recall1	Chinese Taiwan	AD (40)/HC (40) MCI (30)/HC (30)	-	-
			The Wallet Story from ABCD [32]	recall2	Brazilian Portuguese	MCI (23)/HC (12)	-	-
			The Lucia Story Datasets from BALE [32]	recall3	-	AD (9)/HC (80)	-	-
			The Hungarian MCI-mAD Database [26, 27]	recall4	Hungarian	MCI (48)/HC (36) AD (25)/MCI (25)/HC (25)	Yes	No
			- [33]	recall5	Chinese Taiwan	HC (30)/AD (30)	Yes	No
	PD	Cookie theft	pitt Corpus [34]	SS-PD-CT1	English	HC (244)/MCI (309)	Yes	Yes
			ADReSS [19]	SS-PD-CT2	English	AD (78)/non-AD (78)	Yes	Yes
			ADReSSo [35]	SS-PD-CT3	English	AD (87)/HC (79)	Yes	Yes
			Wisconsin Longitudinal Study (WLS) [36]	SS-PD-CT4	English	AD (115)/HC (839)		
			- [37]	SS-PD-CT5	English	AD (26)/HC (46)	Yes	Yes
			NTUHV dataset [31]	SS-PD-CT6	Chinese Taiwan	40 AD/40 HC//30HC/30 MCI	-	-
		-	- [29]	SS-PD1	Italian	MCI (19)/HC (20)	Yes	-
		-	- [24]	SS-PD2	Italian	eD (16)/MCI (32)/HC (48)	Yes	Yes
		MINI-PGA	MINI-PGA [12]	SS-PD3	Spain	AD (6)/HC (12)	Yes	No
		The Dog Story	The Dog Story from BALE [32]	SS-PD4	Brazilian Portuguese	AD (12)/MCI (12)/HC (82)	No	Yes
		The Cinderella Dataset	The Cinderella Dataset [32]	SS-PD5	Brazilian Portuguese	AD (20)/amnestic MCI(20)/HC (20)	Yes	Yes
Reading	Transcripts Reading		Gothenburg MCI study [38]	Reading	Swedish	AD (25)/HC (30)	Yes	No

The full names of abbreviations can be found in “Abbreviations”

### Semantic verbal fluency tasks

The semantic verbal fluency (SVF) test has high sensitivity and specificity for the diagnosis of AD, so it is widely used to assess language skills, semantic memory, and executive functions of AD patients. During the SVF task, patients were asked to list all names they can remember from a category within one minute, such as animals, vegetables, and locations [20].

#### Animal naming

The subjects were asked to say the name of the animal they can think of as quickly as possible within 60 s and were reminded if they stop. At the end of the 60 s, the total number of animals (NOT including repetitions or non-animal words) were counted as their scores [22].

Lopez-De-Ipina, K. et al. constructed a well-distributed animal naming database called PGA-OREKA, which presents a novel proposal based on automatic analysis of speech and disfluencies aimed at supporting MCI diagnosis [21]. The PGA-OREKA database contains 62 healthy people and 38 MCI patients, and it is a subset of the cohort of the Gipuzkoa-Alzheimer Project (PGA) of the CITA-Alzheimer Foundation which includes 187 healthy people and 38 MCI patients.

#### Vegetable and location naming

Similar to animal naming, in vegetable and location naming tests, subjects were asked to say as many words related to the designated topic as possible within one minute. Chien, Y.W. et al. from National Taiwan University constructed a fluency test database based on the Mandarin_Lu corpus [23]. Mandarin_Lu corpus from DementiaBank contains interview recordings of 52 AD patients [24], Chien, Y.W. et al. selected 30 patients and segmented the first-minute response of the audio data, and then recruited 30 additional healthy subjects to complete vegetable and location naming tasks.

### Spontaneous speech tasks

Spontaneous speech (SS) means speech without responding to a question. Temporal parameters of spontaneous speech have been proven to be able to provide sensitive measures of a subject’s speech and language skills [25]. Several different types of spontaneous language tasks are covered in this review paper: conversation/interview speech, event description, recall story, and picture description.

#### Conversation/interview speech

Through natural language processing and analysis of the subject’s speech obtained from free and simple conversational speech, some vital biological features that reflect early signs of AD can be extracted for early screening.

Lopez-De-Ipina, K. et al. built up a multicultural and multilingual database called AZTIAHO [26], which contains 20 h of video recordings of 50 healthy control and 20 AD patients. The recordings consisted of conversational speech where subjects tell pleasant stories or feelings and interact with each other.

#### Day/life/dream description

During these tests, subjects were asked to spontaneously describe events such as tell about the day yesterday in detail. Gosztolya, G. et al. established the Hungarian MCI-mAD Database [27], which recorded 225 voices of 75 subjects (25 AD, 25 MCI, and 25 HC).

#### Recall story

Subjects were given orally presented stories, reading materials, or films to learn the specific stories. Then they were asked to recall and retell the story spontaneously twice, immediately and in a few minutes, to the examiners without reference to those materials.

The Wallet Story database was collected based on the immediate and late retelling of a memorized story from (Bayles and Tomoeda, 1993), which is the evaluation of the episodic memory, one of a standardized test battery named ABCD (Arizona Battery for Communication Disorders) for the comprehensive assessment and screening of dementia. The Wallet Story database included 23 elders with MCI and 12 healthy aging adults, which had 70 narratives in total.

#### Picture description (PD)

Subjects were asked to look at a picture or a series of pictures that make up a story and describe orally the content in pictures within a limited time. Pictures include the cookie theft (a girl and a boy stealing cookies and a woman washing dishes in the kitchen), the dog story (a boy who hides a dog that he found on the street), the Cinderella story, and so on.

Dementiabank [28] is a multimedia interaction for the study of communication in dementia. Pitt corpus [29], ADReSS database [19], and ADReSSo database [30] are subsets of this database. Pitt corpus mainly included recordings of spoken picture descriptions extracted from participants through the cookie theft picture description from the Boston Diagnostic Aphasia Exam [31], which contained 87 speech recordings in AD patients and 79 speech recordings in healthy controls in the training set, and 71 speech recordings without annotations in the testing set. ADReSS database contained speech samples (WAV format) and transcripts (CHA format) with corresponding MMSE (Mini-Mental State Examination) scores as labels, which included 156 subjects, 108 were for training and 48 were for the test (train:test = 7:3). The ADReSSo database was established after the ADReSS database and included 87 AD patients and 79 HC.

### Reading

#### Transcripts reading

Subjects were given short passages or articles to read aloud and their speeches were recorded. The Gothenburg MCI study was conducted as an experiment with 55 Swedish participants (30 HC and 25 AD) who were instructed by a clinician to read a short passage, consisting of 144 words, as part of their evaluation [32].

### Deep learning techniques

In order to investigate the recent progress of deep learning methods in speech-based AD detection, we list some key information in the selected papers in the table below: linguistic tasks, the distribution of participants for each label in the database, the feature types used in papers, the specific model architecture, the model training strategy, and the best performance (Table 2).

**Table 2:** Deep learning techniques in all included papers

References	Year	Task	Sample	Feature type	Classifier	Pre-train	Evaluation	Metrics	Best Performance
Bertini, F. et al. [33]	2022	SS-PD-CT1	AD (137)/HC (43)	AE	auDeep	Yes	CV	Accuracy	93.30%
Meghanani, A. et al. [34]	2021	SS-PD-CT2	AD (54)/non-AD (54)	TLF	FNN	No	Test	Accuracy	83.33%
Rohanian, M. et al. [35]	2021	SS-PD-CT3	AD (122)/HC (115)	TAF/TLF/DF	biLSTM	No	Test	Accuracy	84%
Shah Syed, M.S. et al. [36]	2021	SS-PD-CT2	AD (72)/non-AD (72)	TAF	LSTM^a^	No	Test	Accuracy	74.55%
Mahajan, P. et al. [37]	2021	SS-PD-CT2	AD (82)/non-AD (82)	TAF/DF/TLF/DeF	CNN+biLSTM^a^	No	Test	Accuracy	72.92%
Meghanani, A [34].	2021	SS-PD-CT2	AD (78)/non-AD (78)	TAF	CNN+LSTM	No	Test	Accuracy	64.58%
Lindsay, Hali et al. [38]	2021	VF4	HC (66)/MCI (66)	LE	SVM	Yes	CV	AUC	
Rodrigues Makiuchi, M. et al. [39]	2021	SS-PD-CT1SS-CVS1	CT1: AD (168)/HC (98) CVS1: AD (49)/MCI (42)/HC (72)	TAF	GCNN	No	CV	Accuracy	
Liu, Z. et al. [40]	2021	SS-PD-CT1	AD (252)/HC (232)	AE	CNN+biLSTM^a^	Yes	CV	Accuracy	82.59%
Wang, N. et al. [41]	2021	SS-PD-CT3	AD (87)/HC (79)	TLF/LE	C-Attention-Unified model	Yes	Test	Accuracy	80.28%
Bertini, F. et al. [42]	2021	SS-CVS2SS-PD2	eD (16)/MCI (32)/HC (48)	TAF	FNN	Yes	CV	Accuracy	90.57%
Roshanzamir, A. et al. [43]	2021	SS-PD-CT1	AD (170)/HC (99)	LE	LR	Yes	CV	Accuracy	88.08%
Saltz, P. et al. [44]	2021	SS-PD-CT2SS-PD-CT1	CT2: AD (78)/non-AD (78)	LE	BERTXLNet	Yes	CV	Accuracy	
Liu, Z. et al. [45]	2021	SS-PD-CT2	AD (87)/non-AD (79)	TLF/TAF/LE	BERT	Yes	CV	Accuracy	97.18%
Guo, Y. et al. [46]	2021	SS-PD-CT2SS-PD-CT4	CT2: AD (78)/non-AD (78) CT4: AD (115)/HC(839)	LE	BERT	Yes	Test	Accuracy	82.10%
Pan, Y. et al. [47]	2021	SS-PD-CT2	AD (78)/non-AD (78)	LE	BERT large	Yes	Test	Accuracy	84.51%
Chlasta, K. et al. [48]	2021	SS-PD-CT2	AD (78)/non-AD (78)	AE	DemCNN	Yes	Test	Accuracy	62.50%
Gauder, L. et al. [49]	2021	SS-PD-CT2	AD (87)/non-AD (79)	AE	CNN	Yes	Test	Accuracy	78.90%
Haulcy, R. et al. [50]	2021	SS-PD-CT2	AD (78)/non-AD (78)	LE	SVM, RF	Yes	Test	Accuracy	85.40%
Syed, Z.S. et al. [51]	2021	SS-PD-CT2	AD (78)/non-AD (78)	TLF/LE	SVM, LR	Yes	Test	Accuracy	91.67%
Tsai, A.C. Y. et al. [52]	2021	SS-Recall1 & SS-PD-CT6SS-PD-CT1	SS-Recall1 & SS-PD-CT6 : AD (40)/HC (40)CT1: AD (257)/HC (242)	LE	BERT	Yes	Test	Accuracy	
Zhu, Y. et al. [53]	2021	SS-PD-CT2	AD (78)/non-AD (78)	AE/LE	Longformer	Yes	Test	Accuracy	89.58%
Aparna Balagopalan et al. [54]	2021	SS-PD-CT2	AD (78)/non-AD (78)	LE	BERT	Yes	Test	Accuracy	83.32%
Yuan, J. et al. [55]	2021	SS-PD-CT2	AD (78)/non-AD (78)	LE	ERNIE-large	Yes	Test	Accuracy	89.60%
Xue, C. et al. [56]	2021	SS-CVS9	dementia (330)/MCI (451)/HC (483)	TAF	LSTM	No	CV	Accuracy	67.50%
Roozbeh, S. et al. [57]	2021	SS-PD-CT5	AD (26)/46 (HC)	TAF/TLF	FNN	No	CV	Accuracy	93.05%
Koo, J. et al. [58]	2020	SS-PD-CT2	AD (78)/non-AD (78)	TAF/TLF/AE	CNN+biLSTM^a^	Yes	Test	Accuracy	81.25%
Cummins, N. et al. [59]	2020	SS-PD-CT2	AD (54)/non-AD (54)	TAF/LE	biLSTM^a^	Yes	Test	Accuracy	85.20%
Sarawgi, U. et al. [60]	2020	SS-PD-CT1 SS-PD-CT2	CT1: AD (168)/HC (99)CT2: AD (78)/non-AD (78)	TLF/TAF	FNN	No	CV Test	Accuracy Accuracy	
La Fuente Garcia, S. D. et al. [61]	2020	SS-PD-CT1SS-CVS3	CT1: AD (82)/HC (82) CVS3: AD (30)/HC (16)	TAF	FNN	No	Test	UAR	
Lopez-De-Ipina, K. et al. [62]	2020	VF1	MCI (38)/HC (62)	TAF	CNN	No	CV	Accuracy	92%
Casanova, E. et al. [63]	2020	SS-Recall2SS-Recall3SS-PD4SS-PD5	AD (41)/MCI (55)/HC (194)	TLF	RNN+CRF^a^	Yes	CV	F1-score	81.00%
Pan, Y. et al. [64]	2020	SS-CVS4	ND (21)/MCI (24)/HC (25)	AE	LRSVM	Yes	CV	F1-score	
Searle, T. et al. [65]	2020	SS-PD-CT2	AD (78)/non-AD (78)	LE	DistilBERT	Yes	Test	Accuracy	81%
Li, Y [66].	2020	SS-PD-CT1	AD (155)/HC (145)	DeF/LE/TLF/TLF	LR	Yes	CV	Accuracy	91.25%
Rosas, D.S. et al. [14]	2019	SS-CVS3	Dementia (62)/HC (160)	TLF	FNN	No	Test	Accuracy	86.42%
Chien, Y.W. et al. [67]	2019	SS-Recall5	AD (30)/HC (30)	TAF	biLSTM	Yes	Test	AUC	83.80%
Fritsch, J. et al. [68]	2019	SS-PD-CT1	AD (168)/HC (98)	TLF	LSTM	No	CV	Accuracy	85.60%
Hong, S.Y. et al. [69]	2019	SS-PD-CT1	AD (169)/HC (99)	LE	RNN^a^	Yes	CV	Accuracy	83.50%
Gabor, G. et al. [27]	2019	SS-CVS5SS-Recall4	mAD (25)/MCI (25)/HC (25)	TLF/DF/DeF	SVM	Yes	CV	Accuracy	86.00%
Themistocleous, C. et al. [70]	2018	Reading	HC (30)/MCI (25)	TAF/DeF	FNN	No	CV	Accuracy	83%
Klumpp, P. et al. [71]	2018	SS-PD-CT1	AD (168)/HC (98)	LE	FNN	No	Test	Accuracy	84.40%
Lopez-De-Ipina, K. et al. [72]	2018	VF1SS-CVS6SS-PD3	VF1: MCI (38)/HC (62)CVS6: AD (20)/HC (20)PD3: AD (6)/HC (12)	TAF	CNN	No	CV	Accuracy	
Orimaye, S. O. et al. [73]	2018	SS-PD-CT1	AD task: AD (99)/HC (99) MCI task: MCI (19)/HC (19)	TLF	D2NNLM-5n	No	Test	AUC	
Warnita, T. et al. [74]	2018	SS-PD-CT1	AD (169)/HC (98)	TAF	GCNN	No	CV	Accuracy	73.60%
Chien, Y. W. et al. [23]	2018	VF2VF3	AD (30)/HC (30)	TAF	biLSTM	Yes	Test	AUC	95.40%
Lopez-de-Ipina, K. et al. [12]	2017	VF1SS-CVS6SS-PD3	MCI (40)/HC (60)	TAF	CNN	No	CV	Accuracy	
Lopez-de-Ipina, K. et al. [21]	2017	VF1	MCI (38)/HC (62)	TAF	CNN	No	CV	Accuracy	75%
D Beltrami et al. [75]	2016	SS-CVS7 SS-PD1	MCI (19)/HC (20)	TLF/TAF	FNN	No	CV CV	F1-score F1-score	
Laszlo, T. et al. [76]	2016	SS-CVS5SS-Recall4	MCI (48)/HC (36)	DF/DeF	SVM	Yes	CV	Accuracy	88.10%
Laszlo, T. et al. [16]	2015	SS-CVS8	MCI (32)/HC (19)	TAF/DF	SVM	Yes	CV	Accuracy	80.40%
Lopez-de-Ipina, K. et al. [26]	2013	SS-CVS6	AD (20)/HC (20)	TAF/DF	FNN	No	CV	Accuracy	94.60%

^a^ in Classifier means attention-based method. The full names of abbreviations can be found in “Abbreviations”

### Feature types

Feature types mentioned in our paper include demographic features (DeF), duration features (DF), traditional acoustic features (TAF), traditional linguistic features (TLF), acoustic embeddings, and linguistic embeddings. Demographic features include age, years of education, and gender. Duration features contain the duration of the speaker speaking and its statistics. Traditional acoustic features include properties of the sound wave (MFCCs or Formant), speech rate, and the number of pauses. Traditional linguistic features include lexical (word rate or types and their characteristics, e.g., word frequency, repetitions), semantic (word meaning, e.g., idea density), and syntactic (grammar of sentences, e.g., syntactic complexity, grammatical constituents) features. Acoustic embeddings (AE) means the feature vector representations of speech, which can be extracted by ASR models or pre-trained models (such as speech BERT or YAMNet). Linguistic embeddings (LE) are a type of automatic feature that refers to the vector representations corresponding to input tokens, which can be obtained by models such as BERT [15], ERNIE [77], or Longformer [78].

### Model architectures

In this paragraph, we briefly introduce some deep learning models used in the selected papers, and the model structure used in each paper can be viewed in the table.

#### Feedforward neural network

Earlier researchers started to use feedforward neural networks (FNN) [79] as feature classifiers in their studies to distinguish healthy people from cognitively impaired patients.

#### Convolution neural network

As the convolutional neural network (CNN) [80] has achieved good results in computer vision tasks, CNN-related models have also begun to be gradually applied to NLP tasks, such as sentence classification, semantic parsing, search query retrieval, and other traditional NLP tasks. Therefore, researchers also began to use CNN models and linear gated convolution neural network (GCNN) [81] to classify speech or text data of AD patients.

#### Recurrent meural network

In order to add timing information from speaker audio to the model, researchers began to use model architectures including recurrent neural network (RNN) [82], long short-term memory (LSTM) [83], gated recurrent unit(GRU) [84], bidirectional LSTM(BiLSTM) [85], etc. At the same time, researchers also combine these models with CNN or other neural networks, such as pyramidal bidirectional LSTM followed by a CNN layer (pBiLSTM-CNN) proposed by Meghanani. A [86].

#### Attention-based neural network

With the rise of attention mechanisms [87], researchers began to apply some attention mechanisms to improve the accuracy of the model, such as adding attention mechanisms to RNN models or CNN and LSTM models.

To identify AD with a small amount of data, researchers utilize models pre-trained on large-scale databases as feature extractors to obtain better representations, such as Longformer, BERT, and ERNIE.

## Conclusions

### What were the characteristics of the databases involved in reported studies?

Twenty-seven different databases were used in 52 studies, in which the appearance frequency of the Pitt corpus and ADReSS database were highest. Fourteen studies used Pitt corpus from Dementiabank, and 19 studies included the ADReSS database.

In 27 databases, 11 languages were used. Twenty-five databases used only one language in one database, including Spain, Chinese, English, Hungarian, Italian, Japanese, Brazilian Portuguese, and Swedish. Two databases used more than one language in one database. For example, AZTIAHO included English, French, Spanish, Catalan, Basque, Chinese, Arabian, and Portuguese.

In 29 databases, labels include AD (Alzheimer’s disease), MCI (mild cognitive impairment), and HC (healthy control). Eleven databases contain only AD and HC labels; 7 databases contain only MCI and HC labels; 11 databases contain AD, MCI, and HC labels.

For now, the databases in reported studies were small in single or few languages with uneven distribution. Besides, most were built for cross-sectional studies rather than cohort studies.

### What deep learning model architectures were included in reported studies?

**Fig. 3:**
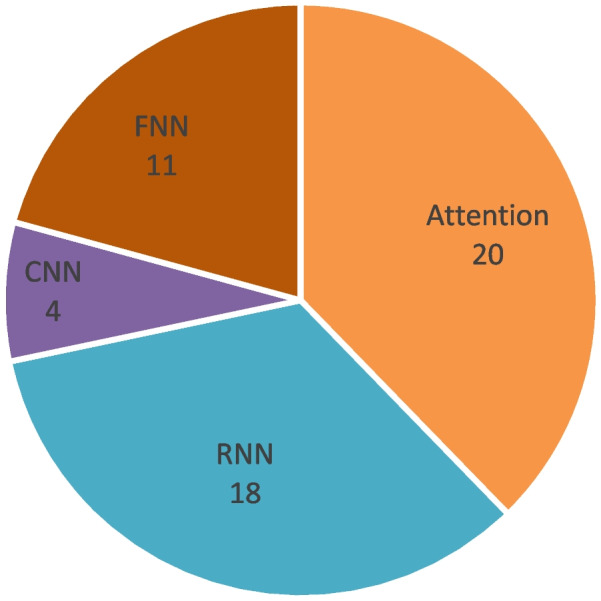
Paper numbers of FNN, CNN, LSTM, and attention mechanism-based models

Four deep learning methods were applied in these selected papers: FNN, CNN, LSTM, and attention mechanism-based models. Figure 3 shows each number of these methods. These models were generally basic, and embeddings were extracted by models and collected for classification.

### How were these deep learning model architectures used in reported studies?

*The use of deep learning* can be divided into three categories. First, the models trained on the large database were directly used to extract embedding, and then machine learning classifiers were used. Second, the models were pre-trained on a large database and then fine-tuned on dementia-related databases. In some situations, Self-training and data augmentation methods were used in the pre-trained process. Thirdly, deep learning models were built and trained from scratch using dementia-related databases.

### What classification performance has been achieved?

#### The performance advantages of deep learning compared to the traditional method

Balagopalan, A. et al. tested on the ADReSS dataset using different classification models, including SVM, NB, RF, FNN, and BERT. According to the results presented in the paper, when using the FNN method, it can achieve an average accuracy of 77.08% on the ADReSS test set in 3 runs, which is higher than the performance of RF and NB but lower than the average accuracy of 81.25% for the SVM classifier. However, when using BERT, it got the best result for classification with an accuracy of 83.32% [54]. Not only linguistic features, but deep learning has also achieved better results on acoustic features. Bertini, F. et al. used an autoencoder to extract unsupervised features from audio data and then utilized FNN to achieve 93.3% classification accuracy on the Pitt dataset, which is better than the results obtained by traditional machine learning methods such as SVM, NB, and RF [33].

In the detection process of AD, utilizing deep learning methods can effectively improve the performance of the classification models when compared with traditional machine learning methods.

**Fig. 4:**
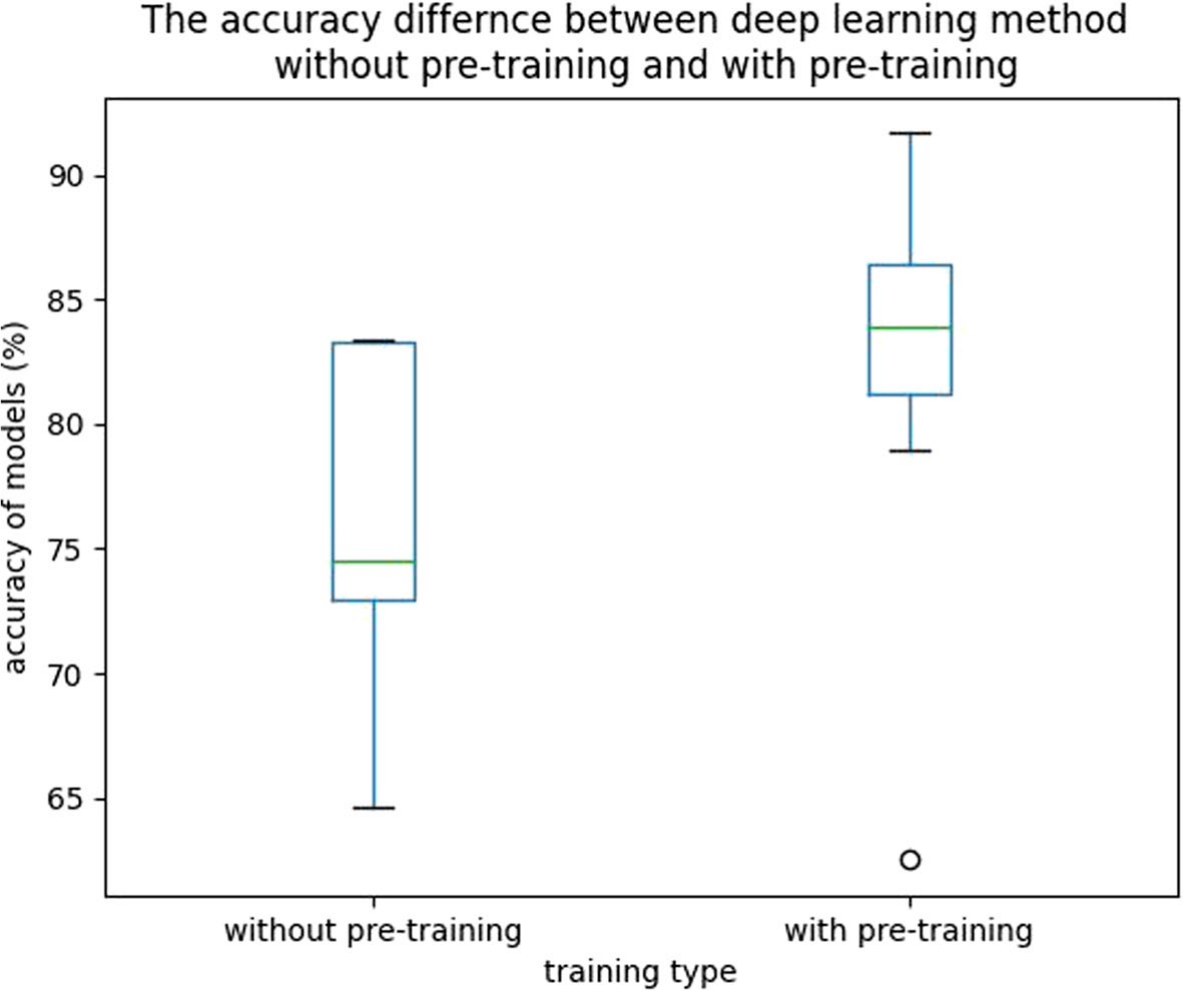
Comparison of deep learning methods without and with pre-training

Besides, we compared methods without pre-training and methods with pre-trainig by box plotting in SS-PD-CT2 task with a test set for evaluation in Fig. 4. It exhibits that using the pre-training method is more useful than training models from scratch.

#### Performance difference based on different tasks

On the task selection, SS works better than others tasks generally. In 2017 and 2018, Lopez-De-Ipina, K. et al. conducted research on AD detection based on VF and SS tasks, in which acoustic features were mainly used. The detection accuracies on SS tasks were higher than the result on the VF task [72].

SS tasks can be divided into several different subtasks, including PD, Conversation/interview, and Recall.

In PD tasks, most tasks were based on ADReSS or Pitt database. There were 21 studies that used the ADReSS database and that 11 studies used the Pitt database. The test set on ADReSS database was uniform, detection accuracy in more than 75% of studies can reach more than 80%, and the best result can reach 91.67%. Cross-validation predictions from 85% of studies on the Pitt database exceeded 80% accuracy, and the best result can reach 91.25%. Ten reported studies contain conversation tasks [14, 16, 26, 27, 39, 42, 56, 64, 75, 76].

Though different databases were used, high accuracy can be achieved by cross-validation evaluation, in which 85% of studies exceeded 85% accuracy and the best result can reach 95%.

In Recall tasks, four related studies are included, and all can achieve 80% accuracy.

#### Comparisons of methods for the ADReSS Challenge

The ADReSS Challenge is the most recent internationally representative speech-based AD detection competition, which was held in Interspeech 2020–2021. The main objective of the ADReSS challenge is to make available a benchmark dataset of spontaneous speech, which is acoustically pre-processed and balanced in terms of age and gender, defining a shared task through which different approaches to AD recognition in spontaneous speech can be compared. Pre-training methods are mainly used in the top five participating teams of the ADReSS challenge, which include two types of useful ways of deep learning techniques.

The first way is pre-training based on deep learning architecture and large datasets, and then fine-tuning on the ADReSS dataset. Saltz, P. et al. [44]; Yuan, J. et al. [55]; and Zhu, Y. et al. [53] used BERT, ERNIE, Longformer-based model architecture to pre-train and then fine-tune, which reached 90%, 89.6%, and 89.58% on ADReSS test set respectively. In terms of characteristics, Saltz, P. et al. and Yuan, J. et al. used linguistic embedding only, and Zhu, Y. et al. used acoustic and linguistic embedding. Besides, Saltz, P. et al. used augmented data during the training stage, Yuan, J. et al. encoded the pause into the transcript and then acquired embedding vector for classification, and Zhu, Y. et al. used Longformer-based transfer learning.

The second way is extracting features based on deep learning architecture, and then training traditional machine learning classifiers based on the extracted features. Syed, Z. S. et al [51] combined traditional linguistic features and linguistic embedding extracted from a pre-trained BERT-based model, and then trained through ensemble learning and fused based on majority-voting, eventually reaching 91.67% accuracy on the ADReSS test set. Haulcy, R. et al. [50] extracted linguistic embedding from BERT with SVM or RF classifier and achieved 85.4% accuracy.

In addition, some other text-based pre-trained models work well. For example, the accuracies of BERT, part of BERT or BERT-based adaptation models [46, 47, 54, 65] were between 81% and 84.51%. Except for the text-based pre-trained models, audio and image-based pre-trained models also have been explored in speech-based AD detection. Chlasta, K. et al [48] trained modified VGGNet architecture to extract acoustic embedding, while Gauder, L. et al. [49] trained wav2vec 2.0 framework to extract acoustic embedding vector, of which both added modified CNN modules for classification, reaching 62.5% and 78.9% accuracy, respectively.

Another training method in the ADReSS Challenge is training from scratch. Traditional linguistic and acoustic features have been applied with the architectures such as FNN [34, 60], attention mechanism-based LSTM [86] and CNN-LSTM [36] model reached 83.33%, 64.58%, and 74.55% accuracy, respectively. After the duration features were added, BiLSTM with highway layers, CNN-BiLSTM-attention-based architecture [35], and dense layer with GRU model [37] reached 84%, 84%, and 72.92% accuracy, respectively.

When using limited clinical data, choosing proper pre-trained task and fine-tuned models are important and effective for disease classification. Generally, CNN-based architectures extract local information, and the LSTM or BERT-based model extracts temporal information. Specifically, pre-training a speech or text encoder with a large speech or text corpus, and using the attention mechanism to map the correspondence, then a fine-tuning model with AD or MCI dataset is a general method to build a framework to train the AD classification from scratch.

#### The algorithms and performances for detecting MCI

As an intermediate transition state between the normal aging process and mild AD, MCI plays an important role in early screening or AD. Among the screened papers, 16 of them performed MCI detection experiments. 11 of the 16 papers were about distinguishing MCI and healthy people, while the rest were about three classifications of AD, MCI patients, and cognitive normal elders.

For the classification of MCI versus cognitive normal subjects, Lindsay, Hali et al. [38] utilized three different pre-trained models (FastTest, Spacy, Wiki2Vec) to extract word embeddings, then used a SVM classifier to predict labels in different languages (French, German, Dutch), and can achieve 66%, 68%, and 69% AUC, respectively. For three-classification experiments for AD, MCI, and HC, Rodrigues Makiuch, M. et al. [39] using a gated convolutional neural network (GCNN), achieving an accuracy of 60.6% in 40 s of speech data.

MCI manifests as mild cognitive decline. Compared with AD, most MCI patients have less severe memory loss and perform relatively normal on memory tests. As can be seen from the papers we screened, it is more difficult to detect MCI patients than to distinguish AD patients from cognitive normal elders-based speech analysis. And we can find that there are not many studies on MCI detection at present, so it is of great value to further explore the methods of detecting MCI with deep learning techniques.

### What were the mainstreams and limitations of reported studies?

The mainstreams and limitations of these selected studies were mainly reflected in language tasks, data modalities, extracted features, and model performance.

#### Language tasks

Varied databases were built to collect speech from AD and healthy people based on varied tasks. Through the databases we introduced in section 4.2 of this article, we can find that the current mainstream language tasks focus on: Semantic verbal fluency tasks, Spontaneous speech tasks, and some other reading tasks.

Semantic verbal fluency tasks contain animal naming tasks, vegetable, and location naming tasks. As for tasks collecting spontaneous speech, it compromised speech from interviews or conversations speech, recall tasks, and picture description tasks.

From this, we can find that there are many kinds of language tasks, which makes it difficult for researchers to compare their research results.

Therefore, based on the picture description task, the Pitt corpus and the ADReSS database have constructed comparable distribution-balanced databases, and researchers have begun to focus on these two databases for AD classification tests.

However, the languages of Pitt corpus and ADReSS databases are both English, and the amount of data is small, so the current research is also limited to a certain extent.

#### Data modalities

Based on our table in the “Deep learning techniques” section, we can see that researchers used speech, text, or speech and text to conduct experiments, in which some compared the classification results on the same evaluation test set.

The current research trend is to obtain more characteristic information by combining multimodal data. Different modalities have different representations, so there is some overlap and complementarity of information, as well as a variety of information interactions. Researchers may no longer be limited to the speech and text information of AD patients. Improving the accuracy of the overall decision-making results by integrating multi-modal data such as eye movement data, writing data, and gait performance is also an interesting topic that needs further investigation.

#### Extracted features

Traditional linguistic and acoustic features were mostly from handcrafted definitions thus these features were explainable. Deep learning-based feature extraction or classification techniques achieved high accuracy for AD classification but short of the lack of interpretability.

Deep learning-based feature extraction methods need a large scale of data, which is hard to precisely define and varies on a different scale of data. Besides, tasks were chosen to pre-train the model for features extraction, for example, ASR or BERT, were not fully compared and analyzed for AD classification tasks.

#### Model performance

How were these deep learning model architectures used in reported studies? and What classification performance has been achieved? In this paper, the deep learning model architectures and training strategies adopted by the selected papers are presented. In the current study, the researchers use the pre-training model to solve the problem of insufficient training data in AD detection and achieve good results. Most speech-based AD detection using deep learning methods can achieve an accuracy of about 85%. In the ADReSS challenge, some researchers have achieved an accuracy of nearly 90% using pretrained models. However, traditional cognitive impairment screening scales, such as MMSE or MOCA, can usually achieve a screening accuracy of more than 93% [5]. Therefore, as a more convenient AD detection method, speech-based deep learning technology needs to be further improved.

## Discussions

### Limitation of our studies

In this review, the following limitations may down the outcome confidence level of our paper:

1. In the process of paper search, our search keywords are missing “pre-trained model,” which leave out some papers that refer to “pre-trained model” but do not mention “deep learning” or “neural network”. Although we add some papers from other sources, this problem increases the risk of bias of the paper search results.
2. Because of our selection criteria, only papers written in English were selected, which resulted in some non-English databases and studies not being included in this review, thus increasing the language bias and affecting some language-related features.
3. Due to the overlap of deep learning methods in many papers, for example, the classifier proposed by Liu, Z. et al. is a combination of CNN, BiLSTM, and attention, so it is difficult to separate it into a specific deep learning category [40]. The lack of a very clear standard in the process of classifying deep learning methods also increases the error of statistical analysis to a certain extent.
4. In the process of analyzing the performance of deep learning models, there may be some potential risks of bias. Because we were only focused on the best performance of the model in the paper, different databases, different testing methods, and different evaluation indicators may possibly lead to a skewed understanding that how well the algorithms worked.

### Research directions

The purpose of this review paper is to investigate current researchers’ application of deep learning methods for speech-based AD detection and to explore future possibilities. The current dementia-related databases are usually small, with a single language, uneven distribution, and inconsistent tasks. However, fusing the multi-modal data rather than using only one modality can extract more useful information for the classification of AD patients, and the application of pre-trained models can also greatly improve the classification accuracy. Another point to note is that the databases in the papers we screened lack cohort study data, so it is difficult to prove the reliability of the results of speech analysis on intra-individual repeated testing. Besides, currently, speech-based AD detection has not been widely applied clinically.

So our future goals are as follows:

1. To establish and publish a balance-distributed Chinese AD database, including the speech data of the picture-distribution task and the writing data of the clock-drawing test.

At the same time, we hope researches can collect cohort data to study the tracking performance of speech analysis in individual patients over time.

(2) To explore the potential of new deep learning models to improve classification accuracy by utilizing speech, writing, and other multi-modal data.

Improving the interpretability of feature representations that have been extracted by deep learning methods in the assessment of cognitive impairment.

(3) To establish efficient and accurate computer-aided diagnosis methods, which can shorten the time of large-scale AD screening. The study on AD detection also promotes the development of portable diagnostic devices, which could timely detect AD and timely intervene to delay the disease.
(4) In addition to Alzheimer’s disease, there are other causes of dementia, so we hope that future researchers can use speech analysis to detect other types of dementia.

## Data Availability

Not applicable.

## Abbreviations

AD: Alzheimer’s disease
MCI: Mild cognitive impairment
HC: Healthy control
ND: Neurodegenerative disorders
mAD: Mild Alzheimer’s disease
eD: Early dementia
MRI: Resonance imaging measurement
PGA: Gipuzkoa-Alzheimer Project
SVF: Semantic verbal fluency
SS: Spontaneous speech
PD: Picture description
TLF: Traditional linguistic features
TAF: Traditional acoustic features
DeF: Demographic features
DF: Duration features
LE: Linguistic embeddings
AE: Acoustic embeddings
CRF: Conditional random field
CV: Cross-validation
UAR: Unweighted average recall
AUC: Area under curve
DNN: Deep neural network
GMM: Gaussian Mixture Model
FNN: Feedforward neural network
SVM: Support vector machine
LR: Logistic regression
RF: Random Forest
ASR: Automatic speech recognition
NLP: Natural language processing
BERT: Bidirectional Encoder Representations from Transformers
ERNIE: Enhanced Representation through kNowledge IntEgration
CNN: Convolutional neural network
GCNN: Gated Convolution Neural Network
GRU: Gated recurrent unit
LSTM: Long short-term memory
RNN: Recurrent neural network
BiLSTM: Bidirectional LSTM
DemCNN: Custom audio convolutional neural network
pBiLSTM-CNN: Pyramidal bidirectional LSTM followed by a CNN layer
D2NNLM-5n: Deep neural network and deep language models with decomposed 5-gram feature
